# Modeling biological systems with uncertain kinetic data using fuzzy continuous Petri nets

**DOI:** 10.1186/s12918-018-0568-8

**Published:** 2018-04-24

**Authors:** Fei Liu, Siyuan Chen, Monika Heiner, Hengjie Song

**Affiliations:** 1 School of Software Engineering, South China University of Technology, Guangzhou, 510006 People’s Republic of China; 2 Control and simulation center, Harbin Institute of Technology, Harbin, 150080 China; 3 Computer Science Institute, Brandenburg University of Technology, Cottbus, 10 13 44 Germany

**Keywords:** Fuzzy uncertainties, Fuzzy continuous Petri nets, Uncertain kinetic parameters, Fuzzy simulation

## Abstract

**Background:**

Uncertainties exist in many biological systems, which can be classified as random uncertainties and fuzzy uncertainties. The former can usually be dealt with using stochastic methods, while the latter have to be handled with such approaches as fuzzy methods.

**Results:**

In this paper, we focus on a special type of biological systems that can be described using ordinary differential equations or continuous Petri nets (CPNs), but some kinetic parameters are missing or inaccurate. For this, we propose a class of fuzzy continuous Petri nets (FCPNs) by combining CPNs and fuzzy logics. We also present and implement a simulation algorithm for FCPNs, and illustrate our method with the heat shock response system.

**Conclusions:**

This approach can be used to model biological systems where some kinetic parameters are not available or their values vary due to some environmental factors.

## Background

Modeling and simulation is one of the main techniques that are used to study biological systems from a computational point of view [1–3]. It plays an essential role in understanding the mechanisms of biological systems and for making predictions for new biological experiments.

So far, a variety of approaches have been proposed for modeling different types of biological systems. These approaches generally can be classified in the following three categories: (1) qualitative approaches such as Boolean networks [4], fuzzy rules [5] and (qualitative) Petri nets [6]; (2) quantitative approaches such as differential equations, Bayesian networks [7] and stochastic/continuous Petri nets [8]; and (3) hybrid approaches by combining qualitative and quantitative methods such as fuzzy stochastic Petri nets [9]. It is natural to employ quantitative (qualitative) approaches when quantitative data are (not) available. However, for many biological systems, not all required quantitative data can be measured completely and precisely, so some kinetic parameters cannot be accurately estimated. In such a case, if we confine ourselves to qualitative methods, any available quantitative data become useless; however if we adopt quantitative methods, some kinetic data are missing or inaccurate. In order to make full use of all the available data for a biological system, hybrid methods could be a good option.

The uncertainties in biological systems usually come from their intrinsic internal noises and external environmental factors, or are caused by measurements. These uncertainties can be further classified according to their sources in two categories: random uncertainties and fuzzy uncertainties. If there are insufficient or missing data for a biological system, the modeling of the system could be accompanied with fuzzy uncertainties. Random uncertainties usually can be dealt with using stochastic methods such as stochastic Petri nets or stochastic differential equations, while fuzzy uncertainties have to be handled with such methods as fuzzy methods.

In this paper, we focus on a class of biological systems, which can be described using ordinary differential equations (ODEs) or continuous Petri nets (CPNs) [10], but have some inaccurate or missing kinetic parameters. Because of the existence of fuzzy uncertainties caused by insufficient data, we may have to combine fuzzy methods with quantitative methods such as ODEs to accomplish a trustworthy modeling of such a class of biological systems. In order to achieve this, we are going to propose a class of fuzzy continuous Petri nets (FCPNs).

Petri nets have been widely used in systems biology these years and have been extended in many ways, e.g., stochastic Petri nets by associating a stochastic time delay with each transition [11], and continuous Petri nets (CPNs) by associating deterministic rates with transitions and allowing tokens on places to be real values [12]. The underlying semantics of a CPN is a set of ODEs, which means a CPN model is nothing else than a graphical representation of a set of ODEs. By doing so, biologists can easily construct biological models described by ODEs and the constructed models are less error prone. On the other hand, fuzzy logic [13] was proposed to deal with fuzzy uncertainties, and has been applied in many fields. Fuzzy logic has also been combined with differential equations (DEs), and different types of fuzzy DEs have been proposed [14, 15]. This offers good means to cope with quantitative systems involving uncertainties.

In this paper, we aim at the modeling of uncertain biological systems, and propose a class of biologically interpreted FCPNs by allowing some kinetic parameters to be fuzzy numbers. Considering the fact that analytical methods are impossible for large models [14], we present an appropriate algorithm for simulating large FCPNs. We illustrate the application of our method with a medium-sized model.

We believe that our approach can be applied in the following biological scenarios.

- For a biological model, if some of its kinetic parameters are not available, thus precluding ODE simulation, we can still use FCPNs to quantitatively analyze the model by giving an uncertain band of any output, rather than crisp values.
- For a biological model where some of its parameters vary due to environmental effects or other factors and stochastic methods are not appropriate, then we can represent each variable (or uncertain) parameter as a fuzzy number. By running fuzzy simulation, we can obtain an uncertain band of an output, which describes the effect of the variable parameters.

Taking into account the fact that many ODE and CPN models of biological systems do exist and intrinsic uncertainties are associated with many biological systems, we believe that FCPNs offer a new means for reexamining these existing ODE and CPN models, revealing potentially new insights into the corresponding biological systems.

**Our fuzzy modeling approach vs parameter estimation methods.** Parameter estimation is an essential step in constructing quantitative (biological) models, which aims to infer kinetic parameters from experiment observations [16]. In contrast, our approach is one of the fuzzy modeling approaches, which aims to derive the uncertainties of outputs from the uncertainties of input parameters. In this paper, we will present a workflow for using our approach, where we will clearly see that parameter estimation can be considered as a key step of our fuzzy modeling approach.

The structure of the paper is as follows. In the section of methods, we describe fuzzy sets and continuous Petri nets. In the section of results and discussion, we present a class of fuzzy continuous Petri nets together with a fuzzy simulation algorithm, and discuss how to use the approach for modeling and analyzing biological systems illustrated by a medium-sized biological model. Finally, the conclusions are given.

## Methods

In this section, we introduce fuzzy sets and continuous Petri nets.

### Fuzzy sets

Fuzzy sets, proposed by Zadeh [13], are a generalization of classical sets and can handle uncertainty associated with imprecision and vagueness. Fuzzy theory is different from probability theory that deals with randomness.

A fuzzy set $\tilde {\xi }$ is defined on a universal set $\mathbb {X}$ by its membership function

1 $$ \mu_{\tilde{\xi}}: \mathbb{X} \rightarrow [\!0,1],  $$

which maps a real value $\mu _{\tilde {\xi }}(x) \in ~[\!0,1]$ to each element $x\in \mathbb {X}$. That is, in a fuzzy set, each element has a membership degree between 0 and 1, which is different from any element in a crisp set, whose membership degree is either 0 or 1.

The *α*-cut of a fuzzy set $\tilde {\xi }$ at a level *α*∈ [ 0,1] is defined as the crisp subset of $\mathbb {X}$ with all the elements whose membership degree is greater than or equal to the given *α*, i.e.,

2 $$ \tilde{\xi}_{\alpha} = \{x| \mu_{\tilde{\xi}}(x)\geq \alpha, x\in \mathbb{X}, \alpha \in~ [\!0,1] \}.  $$

**Fig. 1:**
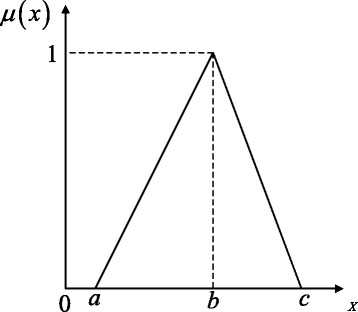
A triangular fuzzy number, $\tilde {\xi }=(a,b,c)$. *a* is only allowed to be greater than 0 for the biological applications

A fuzzy number is a special convex normalized fuzzy set over the real set $\mathbb {R}$. Many different types of fuzzy numbers have been defined such as triangular, trapezoidal and bell-shaped fuzzy numbers. A triangular fuzzy number, denoted by $\tilde {\xi }=(a,b,c)$, *a*≤*b*≤*c*, is defined as (see Fig. 1):

3 $$  \mu_{\tilde{\xi}}(x) =\left\{ \begin{array}{l l} 0 & \quad \text{if \(x \leq a\)}, \\ \frac{x-a}{b-a} & \quad \text{if \(a\leq x \leq b\)},\\ \frac{c-x}{c-b} & \quad \text{if \(b\leq x \leq c\)},\\ 0 & \quad \text{else}. \end{array}\right.  $$

Its *α*-cut for any *α*∈ [ 0,1] is simply written as

4 $$  \tilde{\xi}_{\alpha}=[a+\alpha(b-a), c-\alpha(c-b)].  $$

In the following, we arbitrarily chose to consider triangular fuzzy numbers to illustrate our approach, without loss of generality. We denote by *Γ* the set of triangular fuzzy numbers whose lower bound is greater than 0, i.e. *a*>0.

The extension principle is a powerful tool of fuzzy logic, which offers a general procedure for extending crisp domains to fuzzy domains. Assume $f:{{\mathbb {X}}^{n}}\to \mathbb {Y}$, and $\tilde {A}$ is a fuzzy set on $\mathbb {X}$ such that

$$\tilde{A}=\mu_{\tilde{A}}(x_{1})/(x_{1})+\mu_{\tilde{A}}(x_{2})/(x_{2})+\ldots+\mu_{\tilde{A}}(x_{n})/(x_{n}).$$

Then, applying the extension principle yields the following fuzzy set

$${} \tilde{B}=f(\tilde{A})= \mu_{\tilde{A}}(y_{1})/(y_{1})+\mu_{\tilde{A}}(y_{2})/(y_{2})+\ldots+\mu_{\tilde{A}}(y_{n})/(y_{n}),$$

where $x_{i} \in \mathbb {X}$, $y_{i} \in \mathbb {Y}$, *y*_*i*_=*f*(*x*_*i*_), *i*=1,2,…,*n*.

The extension principle will be used in the paper to achieve fuzzy simulation of FCPNs.

**Fuzzy differential equations (FDEs).** As a typical class of FDEs, a differential equation with fuzzy parameters can be defined as

5 $$  \left\{ \begin{array}{lr} dx/dt = f(t,x(t),C), \\ x(t_{0})=x_{0}, \end{array} \right.  $$

where *C* is a fuzzy parameter, represented as a fuzzy number.

The analytical solution of Eq. 5 can be obtained via a family of differential inclusions [17], or by considering *dx*/*dt* as the fuzzy generalized derivative [18]. However, all these existing analytical methods are only applicable for one or two equations. In order to address a number of FDEs, numerical methods have to be employed. In this paper, we will present a numerical approach for solving fuzzy differential equations.

### Continuous Petri nets

Petri nets (PNs) [19] are weighted, directed, bipartite multigraphs. A PN consists of places, transitions (both of which are called nodes) and arcs (or edges) that connect nodes of both types. In the biological scenario, places may represent chemical species or their compounds, e.g., genes, mRNA, proteins or protein complexes; transitions may represent any kind of chemical reactions (e.g. association, disassociation, translation or transcription) or any positive/passive behavior such as molecular movement [20, 21]. The tokens on places represent the number of molecules or the concentration levels of species, which only allow discrete integers.

For modeling a variety of scenarios, PNs have been extended in many ways, one of which is continuous Petri nets (CPNs) by allowing tokens on places to be real values to represent the concentration of species. The formal definition of a CPN is given as follows [11].

A CPN is a six-tuple *N*=<*P*,*T*,*F*,*f*,*v*,*M*_0_>, where

- *P* is a finite, non-empty set of continuous places.
- *T* is a finite, non-empty set of continuous transitions.
- *F*⊆(*P*×*T*)∪(*T*×*P*) is a finite set of directed arcs.
- $f: F \rightarrow \mathbb {R}_{0}^{+}$ is a function that assigns a non-negative real number to each arc *a*∈*F*. $\mathbb {R}_{0}^{+}$ denotes the set of non-negative real numbers.
- *v*:*T*→*H* is a function that assigns a firing rate function *h*_*t*_ to each transition *t*∈*T*, whereby $H := \bigcup _{t \in T}\left \{ h_{t}|h_{t}:\mathbb {R}_{0}^{{+}^{|^{\bullet } t|}} \rightarrow \mathbb {R} \right \}$ is the set of all firing rate functions, and *v*(*t*)=*h*_*t*_ for all transitions *t*∈*T*. $\mathbb {R}$ denotes the set of real numbers. ^∙^*t* denotes the preplaces of transition *t*.
- $M_{0}: P\rightarrow \mathbb {R}_{0}^{+}$ gives the initial marking, which assigns a non-negative real number to each place *p*∈*P*.

In a CPN, besides the continuous token values on places, the transitions are also continuous. That is, each transition is associated with a firing rate function and can continuously fire during simulation, if its preplaces allow to do so.

In fact, the underlying semantics of a CPN is a system of ODEs, where each equation describes the continuous token change on a given place, i.e., continuously increasing by its pretransitions’ flow and decreasing by its posttransitions’ flow. An equation for a place *p* takes the following form:

$$\frac{d(p)}{dt} = \sum\limits_{t \in^{\bullet} p} f(t,p)v(t) - \sum\limits_{t \in p^{\bullet}} f(p,t)v(t), $$

where ^∙^*p* and *p*^∙^ denote the pretransitions and posttransitions of place *p*, respectively.

Besides, the rate function related to each transition can be further written in the following form:

6 $$  h(t,\theta):\mathbb{R}_{0}^{{+}^{|^{\bullet} t|}} \rightarrow \mathbb{R},  $$

where *θ* is a rate constant (also called kinetic constant).

**Fig. 2:**
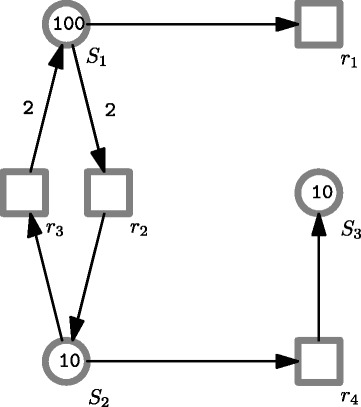
A Petri net model of a decay-dimerization network [26]. This network consists of a degradation reaction (*r*_1_), two reversible dimerization reactions (*r*_2_ and *r*_3_), and a conversion reaction (*r*_4_)

For example, Fig. 2 gives a CPN model of a decay-dimerization network, which includes the following four biochemical reactions:

$$\begin{array}{*{20}l} r_{1}: & \, S_{1} \stackrel{\theta_{1}}{\rightarrow} \emptyset \\ r_{2}: & \, S_{1} + S_{1} \stackrel{\theta_{2}}{\rightarrow} S_{2} \\ r_{3}: & \, S_{2} \stackrel{\theta_{3}}{\rightarrow} S_{1} + S_{1} \\ r_{4}: & \, S_{2} \stackrel{\theta_{4}}{\rightarrow} S_{3} \end{array} $$

This CPN model generates the following set of ODEs under the mass action semantics.

$$\begin{array}{*{20}l} {dS}_{1}/dt = &\, 2*(\theta_{3}*S_{2})-(\theta_{1}*S_{1})-2*\left(\theta_{2}*S_{1}^{2}\right), \\ {dS}_{2}/dt = &\, \left(\theta_{2}*S_{1}^{2}\right)-(\theta_{3}*S_{2})-(\theta_{4}*S_{2}),\\ {dS}_{3}/dt = &\, (\theta_{4}*S_{2}). \end{array} $$

**Fig. 3:**
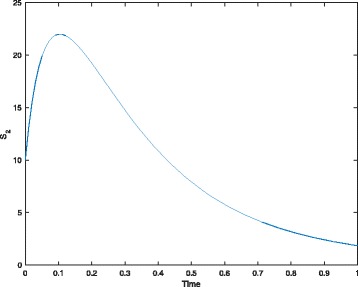
A simulation plot of the CPN model in Fig. 2. Parameters: *θ*_1_=0.2, *θ*_2_=0.04, *θ*_3_=0.5 and *θ*_4_=5

If we run numerical simulation of the CPN model, we obtain the simulation result that is illustrated in Fig. 3 by taking the species *S*_2_ as an example.

## Results and discussion

In this section, we present a class of fuzzy continuous Petri nets together with a fuzzy simulation algorithm, and discuss how to use the approach for modeling and analyzing biological systems illustrated by a medium-sized biological model.

### Fuzzy continuous Petri nets

In Eq. 6, the kinetic constant *θ* is usually obtained using parameter estimation methods. If *θ* is not precise, the CPN model will not produce the correct result. In this case, we have to abandon CPNs or ODEs and turn to other methods able to deal with uncertainties at the cost of losing all available data.

To overcome this issue, we may turn to hybrid methods by replacing the values of those uncertain parameters with fuzzy numbers. Thus, in such a model, we allow that some parameters are crisp, but others fuzzy numbers. In this way, we can make full use of all the available data of a system. In this case, the rate function of each transition turns into the following form (either a real value or a fuzzy number in *Γ*):

7 $$  h(t,\theta):\mathbb{R}_{0}^{{+}^{|^{\bullet} t|}} \rightarrow \mathbb{R} \cup \Gamma.  $$

Formally, an FCPN is defined as a six-tuple *N*=<*P*,*T*,*F*,*f*,*v*,*M*_0_>, where

- *P* is a finite, non-empty set of continuous places.
- *T* is a finite, non-empty set of continuous transitions.
- *F*⊆(*P*×*T*)∪(*T*×*P*) is a finite set of directed arcs.
- $f: F \rightarrow \mathbb {R}_{0}^{+}$ is a function that assigns a non-negative real number to each arc *a*∈*F*.
- *v*:*T*→*H* is a function that assigns a firing rate function *h*_*t*_ to each transition *t*∈*T*, whereby $H := \bigcup _{t \in T}\{ h(t,\theta)\}$ is the set of all firing rate functions, and *v*(*t*)=*h*_*t*_ for all transitions *t*∈*T*, and *h*(*t*,*θ*) in defined by Eq. (7).
- $M_{0}: P\rightarrow \mathbb {R}_{0}^{+}$ gives the initial marking, which assigns a non-negative real number to each place *p*∈*P*.

With FCPNs, we can model *θ* as a crisp value if precise quantitative data are available or as a fuzzy number if the parameter cannot be measured precisely. As a result, we may use fuzzy analytical or fuzzy simulation methods to obtain an uncertain band for each output according to the uncertain band of the parameters. Therefore, we still obtain a quantitative analysis of a biological model that lacks some quantitative data. By presenting FCPNs, we offer a new method for modeling and analyzing biological systems with uncertain information.

The semantics of an FCPN is described by a combination of a set of FDEs in the form of Eq. 5 and a set of ODEs. Due to the existence of a couple of FDEs for a biological model, we have to resort to a simulation approach to analyze an FCPN model. Using the extension principle, we obtain the uncertainty of model outputs according to the uncertainty of model parameters.

**Fig. 4:**
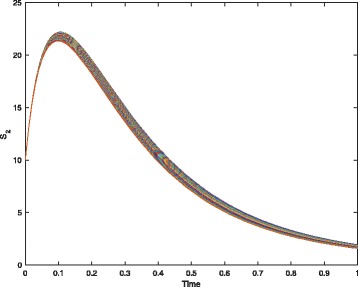
A simulation plot of the FCPN model. See Table 1 for the values of parameters

For example, when we replace the crisp values of parameters *θ*_3_ and *θ*_4_ with the fuzzy numbers given in Table 1, we obtain an FCPN model for the decay-dimerization network, which looks exactly the same as the one in Fig. 2 except of the two fuzzy kinetic parameters. Besides, Fig. 4 gives a simulation plot of the model, which is an uncertain band of an output due to the uncertain parameters. We can check the correctness of the model by analyzing the uncertain band of each output.

**Table 1:** Rate functions of the transitions given in the FCPN model of Fig. 2

Transition *t*	Rate function *h*(*t*,*θ*)	Kinetic constant *θ*
*r*_1_	*θ*_1_∗*S*_1_	0.2
*r*_2_	*θ*_2_∗*S*_1_∗*S*_1_	0.04
*r*_3_	*θ*_3_∗*S*_2_	(0.45,0.5,0.55)
*r*_4_	*θ*_4_∗*S*_2_	(4.9,5.0,5.4)

### Simulation algorithm

A biological model constructed as FCPNs usually generates a number of ODEs and/or FDEs. Analytical methods that are applicable for several simple FDEs can hardly be applied for analyzing such models. Therefore, numerical simulation becomes essential, especially for larger models.

For achieving numerical simulation of an FCPN model, we adopt the following idea. We first represent each fuzzy number as a union of its *α*-cuts according to Zadeh’s extension principle. By sampling the *α*-cut at each *α* level, we obtain a combination of samples for all parameters. For each combination, we run numerical simulation on the corresponding CPN model at an *α* level. After running simulations for all considered *α* levels, we compose all the *α*-cuts to obtain the membership function of each output at each simulation time point. That is, we obtain the uncertainties of outputs caused by the uncertainties of kinetic parameters.

This procedure is given in Algorithm 1 and explained in depth in the following.

**Fig. 5:**
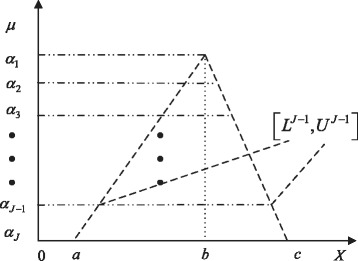
Decompose a membership function into its *α*-cuts for a fuzzy kinetic parameter. This is done according to Zadeh’s extension principle

**Fig. 6:**
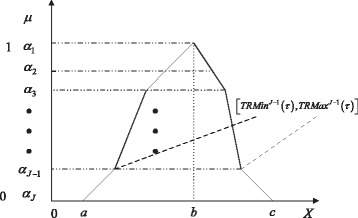
Compose a set of *α*-cuts to form a membership function for an uncertain output at a time point *τ*. This is done according to Zadeh’s extension principle

(1) Determine the appropriate number of *α* levels to decompose the membership functions of uncertain parameters (Line 1). Each level is denoted by *α*_*j*_, *j*=1,2,…,*J*, where *J* is the total number of *α* levels to be considered. Each parameter takes the same number of *α* levels.

(2) For each *α* level, compute the corresponding *α*-cut of each fuzzy number $\tilde {\theta }_{i}$, *i*=1,2,…,*I*, according to Eq. 4, where *I* is the total number of fuzzy numbers contained in the uncertain parameters. Each *α*-cut is represented as $\left (\tilde {\theta }_{i}\right)_{\alpha _{j}} = \left [L_{i}^{j}, U_{i}^{j}\right ]$ (Line 3). Figure 5 illustrates the result by performing the first two steps.

(3) Discretize each *α*-cut $\left [L_{i}^{j}, U_{i}^{j}\right ]$ and obtain a set of crisp values for each fuzzy number (Line 4). To do this, we use the same sampling step for simplicity, but of course we can adopt any other sampling method. But in order to improve the computational efficiency, we have to optimize the sampling or even minimize the sample number. Assume we sample *K* discretization values for each *α*-cut. We then obtain *K*^*I*^ combinations of these sampling values for *I* fuzzy numbers at each *α* level. Thus we have *K*^*I*^×*J* combinations for all *α* levels. That is, the time complexity of the algorithm can be represented as *O*(*K*^*I*^×*J*). For an uncertain model, the number of its uncertain parameters, i.e. *I*, usually can be fixed. Therefore, we should try to decrease both *K* and *J*.

(4) For each combination *c*∈*K*^*I*^×*J*, we replace each fuzzy number with its corresponding sampling value and then obtain a sample (a set of ODEs) of the FCPN model (Line 7). After that, we perform numerical simulation on the corresponding ODEs with a numerical integration method such as the Runge–Kutta method, and obtain simulation results (Lines 8-9).

(5) We compose the simulation traces of all the *α*-cuts to obtain the membership function of each output at each time point, which is illustrated in Fig. 6 (Lines 11-16). Such membership function reflects more accurately the effects of uncertain parameters.

(6) By computing the maximum and minimum values over all the simulation traces at each time point for each output, we obtain an uncertain band for the output (Lines 18-21). Such uncertain band roughly reflects the effects of uncertain parameters.



**A more efficient sampling method.** The sampling method we use above involves a large number of samples, i.e., *K*^*I*^×*J*; however, there may be some redundancy at some *α* levels. By reducing this redundancy without affecting the accuracy, we propose a new sampling method, which works as follows.

For each fuzzy parameter *i*, we only discretize the *α*-cut at *α*_*J*_=0 into *k* crisp values, while for other *α* levels, we only consider the start and end points, i.e. $L_{i}^{j}$ and $U_{i}^{j}$. Thus we have in total *K*^*I*^+2^*I*^×(*J*−1) combinations, which result in the same number of simulation traces, each of which is denoted by *Tr*_*ijk*_ for an output. The new sampling method substantially reduces the number of samples compared with the method above. When the *α* level *m* is equal to *J*, we use the same method as given in Algorithm 1 to compute the membership function and uncertain band of an output. When *m* is unequal to *J*, we first obtain the traces to be used at this *α* level, which consist of the traces in *Tr*_*imk*_ and the others in *Tr*_*iJk*_. The latter can be obtained in the following way. If $v_{iJk} \in \left [L_{i}^{m}, U_{i}^{m}\right ]$ for all fuzzy parameters, where *v*_*iJk*_ is the crisp parameter value via discretation at the *α* level *J*, the corresponding trace *Tr*_*iJk*_ is selected. After that, we still use the same method given in Algorithm 1 to compute the membership function and uncertain band of an output.

### A workflow to use FCPNs for modeling biological systems

In this section, we give a general workflow to use FCPNs to model and analyze biological systems.

(1) Collect quantitative data and qualitative knowledge for the biological system to be studied. A model usually consists of two main parts, structure and kinetic parameters. We first determine the structure of the model by means of the available data and knowledge. At this step, we can obtain a qualitative Petri net model, which can be read as a set of ODEs in which parameters are not assigned values. We then divide the kinetic parameters of the model into two categories: precise or uncertain.

(2) For parameters with sufficient quantitative data, we can use well-established parameter estimation methods to obtain their precise values. See e.g., [22, 23] for performing parameter estimation of biological models described by ODEs.

(3) For each uncertain parameter, we can adopt two methods to specify their fuzzy values. (a) Perform parameter estimation based on the available incomplete quantitative data. When parameter estimation is performed, we usually specify the initial parameter search space and then refine this space based on the data. If the data is insufficient, we cannot obtain a precise parameter value, although we may be able to reduce the search space. In this case, we will use the reduce parameter space to specify the fuzzy value of the parameter. (b) Employ experts to directly give a fuzzy value for a parameter. If quantitative data are not available, we can ask experts to assign a fuzzy number to a parameter according to their experience. By assigning values to all parameters, either crisp or fuzzy, we obtain a complete FCPN model for a biological system.

(4) We then run fuzzy simulation using the algorithm given above to obtain uncertain outputs and analyze them. After the model is validated, we can use it to explain the corresponding biological phenomena and potentially make predictions for further experiments.

### Case study

In this section, we use a heat shock response model given in [24] to illustrate the approach presented in the paper.

#### Modeling

The heat shock response is a highly conserved genetic network that acts as a main defense mechanism against cell stress and protein damage. The biochemical reactions considered in [24] are repeated in Table 2, and the species involved in the model are explained in Table 3 together with their initial values [24].

**Table 2:** The biochemical reactions involved in the heat shock response model, adapted from [24]

Species	Description
*r*_1_(*r*_2_):	$2hsf\underset {{{k}_{1m}}}{\overset {{{k}_{1p}}}{\rightleftharpoons }}hs{{f}_{2}}$
*r*_3_(*r*_4_):	$hsf+hs{{f}_{2}}\underset {{{k}_{2m}}}{\overset {{{k}_{2p}}}{\rightleftharpoons }}hs{{f}_{3}}$
*r*_5_(*r*_6_):	$hs{{f}_{3}}+hse\underset {{{k}_{3m}}}{\overset {{{k}_{3p}}}{\rightleftharpoons }}hs{{f}_{3}}\_hse$
*r*_7_:	$hs{{f}_{3}}\_hse\xrightarrow {{{k}_{4}}}hs{{f}_{3}}\_hse+hsp$
*r*_8_(*r*_9_):	$hsp+hsf\underset {{{k}_{5m}}}{\overset {{{k}_{5p}}}{\rightleftharpoons }}hsp\_hsf$
*r*_10_:	$hsp+hs{{f}_{2}}\xrightarrow {{{k}_{6}}}hsp\_hsf+hsf$
*r*_11_:	$hsp+hs{{f}_{3}}\xrightarrow {{{k}_{7}}}hsp\_hsf+2hsf$
*r*_12_:	$hsp+hs{{f}_{3}}\_hse\xrightarrow {{{k}_{8}}}hsp\_hsf+hse+2hsf$
*r*_13_:	$hsp\xrightarrow {{{k}_{9}}}$
*r*_14_:	$prot\xrightarrow {{{k}_{10}}}mfp$
*r*_15_(*r*_16_):	$hsp+mfp\underset {{{k}_{11m}}}{\overset {{{k}_{11p}}}{\rightleftharpoons }}hsp\_mfp$
*r*_17_:	$hsp\_mfp\xrightarrow {{{k}_{12}}}hsp+prot$

See Table 4 for the values of the kinetic parameters

**Table 3:** The species involved in the heat shock response model

Species	Description	Initial value
*h* *s* *f*	Heat shock factor	0.67
*h* *s* *f*_2_	Phosphorylation state of hsf	8.7×10^−4^
*h* *s* *f*_3_	Phosphorylation state of hsf	1.2×10^−4^
*h* *s* *e*	Heat shock element	29.73
*hsf*_3__*hse*	The binding of *hsf*_3_ to *hse*	2.96
*h* *s* *p*	Heat shock protein	766.88
*hsp*_*hsf*	The binding of *hsp* to *hsf*	1403.13
*m* *f* *p*	The heat-induced misfolded protein	517.352
*hsp*_*mfp*	The binding of *hsp* to *mfp*	71.65
*p* *r* *o* *t*	Unfolded or native protein	1.15×10^8^

The initial values are taken from [24]

**Table 4:** Values of the kinetic parameters, obtained from [24]

Parameter	Value	Unit
*k*_1*p*_	3.49	*ml*/*#s*
*k*_1*m*_	0.19	*s* ^−1^
*k*_2*p*_	1.07	*ml*/*#s*
*k*_2*m*_	10^−9^	*s* ^−1^
*k*_3*p*_	0.17	*ml*/*#s*
*k*_3*m*_	1.21×10^−6^	*s* ^−1^
*k*_4_	8.3×10^−3^	*s* ^−1^
*k*_5*p*_	9.74	*ml*/*#s*
*k*_5*m*_	3.56	*s* ^−1^
*k*_6_	2.33	*ml*/*#s*
*k*_7_	4.31×10^−5^	*ml*/*#s*
*k*_8_	2.73×10^−7^	*ml*/*#s*
*k*_9_	3.2×10^−5^	*s* ^−1^
*k*_10_	$ \left (1-\frac {0.4}{{{e}^{T-37}}} \right)\times {{1.4}^{T-37}}\times 1.45\times {{10}^{-5}}$	*s* ^−1^
*k*_11*p*_	3.32×10^−3^	*ml*/*#s*
*k*_11*m*_	4.44	*s* ^−1^
*k*_12_	13.94	*s* ^−1^

Here *T* is the temperature

The heat shock factor (*hsf*) has two phosphorylation states *hsf*_2_ and *hsf*_3_; these three species can be converted from one to another by phosphorylation or dephosphorylation. The heat shock protein (*hsp*) plays the key role in preventing misfolding and facilitating protein folding. The *hsp*-encoding genes can be transactivated through the binding of *hsf*_3_ to the heat shock element (*hse*), which forms *hsf*_3__*hse*. *hsp* may also bind to *hsf*, forming *hsp*_*hsf*, or bind to *hsf*_2_ or *hsf*_3_. *hsp* can also bind to the heat-induced misfolded proteins *mfp* which are the drivers of the whole heat shock response, forming *hsp*_*mfp*. *mfp* is converted from an unfolded or native protein (*prot*) induced by the heat.

**Fig. 7:**
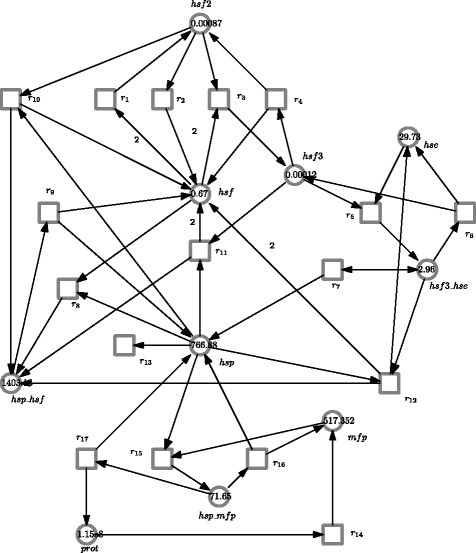
A CPN/FCPN model of the heat shock response. Depending on the form of kinetic parameters, the model can be interpreted as a CPN or FCPN one

According to these reactions, we build a CPN/FCPN model, depending on the type of parameters, illustrated in Fig. 7. From the model, we see that we map each reaction to a transition of the CPN/FCPN model; a reversible reaction is considered as two irreversible reactions. We also assume that the principle of mass action applies to each reaction.

#### Structural analysis

As a member of the family of Petri nets, FCPNs likewise enjoy all Petri net analysis techniques. Here we will analyze T-invariants [6] of our constructed model by feeding the FCPN model given in Fig. 7 to Charlie [25], an analysis tool of Petri net models.

Our model in hand is covered by 10 minimal T-invariants, i.e., {(*r*_1_,*r*_2_), (*r*_3_,*r*_4_), (*r*_5_,*r*_6_), (*r*_8_,*r*_9_), (*r*_15_,*r*_16_), (*r*_1_,*r*_9_,*r*_10_), (*r*_1_,*r*_3_,*r*_9_,*r*_11_), (*r*_1_,*r*_3_,*r*_5_,*r*_9_,*r*_12_), (*r*_14_,*r*_15_,*r*_17_), (*r*_7_,*r*_13_)}. Each T-invariant is an elementary behavioral mode of a system, and reproduces a system state (or marking) [6]. The first four T-invariants correspond to the four reversible reactions given above, while the others reveal further elementary behavioral modes that cannot be easily deduced from the reaction equations. For example, the T-invariant (*r*_7_,*r*_13_) shows that dissociation (of *hsf*_3__*hse*) and degradation (of *hse*) form an elementary behavioral mode, and in fact this kind of mode is a basic and widely-seen biological module. Besides, we can also structure the model using these T-invariants.

#### Simulation analysis

**Fig. 8:**
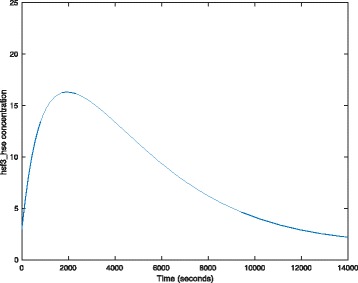
A simulation plot of the CPN model in Fig. 7. See Table 4 for the values of the kinetic parameters

We first validate our model with the parameters given in [24] (see Table 4), and obtain the simulation result. For example, a plot of *hsf*_3__*hse* is given in Fig. 8, which is completely consistent with the one given in [24].

**Fig. 9:**
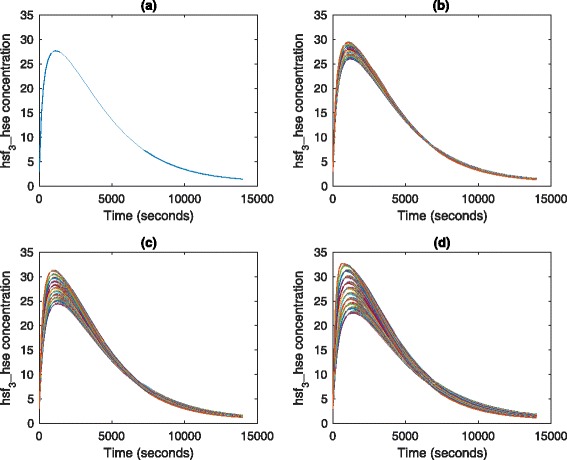
A two-dimensional simulation plot of *hsf*_3__*hse* for the FCPN model in Fig. 7. The two fuzzy numbers are given as follows: *k*_8_=(2.457×10^−7^,2.73×10^−7^,3.003×10^−7^), *T*=(41.8,42,42.2). **a** *α*=1.0, **b** *α*=0.7, **c** *α*=0.4, **d** *α*=0

**Fig. 10:**
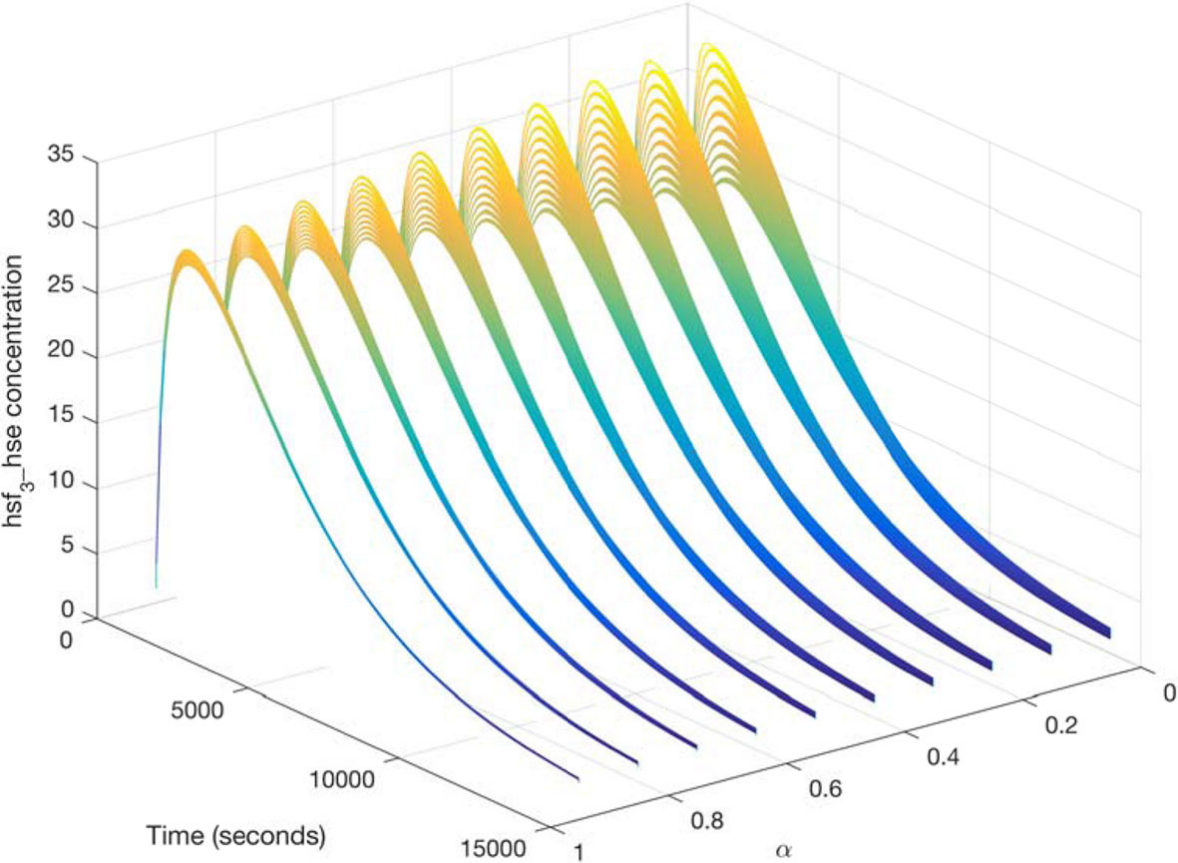
A three-dimensional simulation plot of *hsf*_3__*hse* for the FCPN model in Fig. 7. The same values of the kinetic parameters are used as those given in Fig. 9

**Fig. 11:**
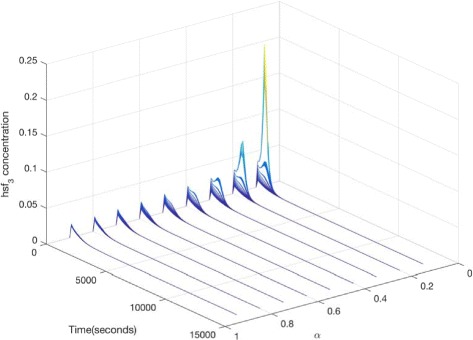
A three-dimensional simulation plot of *hsf*_3_ for the FCPN model in Fig. 7. The same values of the kinetic parameters are used as those given in Fig. 9

We now illustrate our approach by setting the values of parameters *T* and *k*_8_ to fuzzy numbers. For example, a plot of *hsf*_3__*hse* is given in Fig. 9 with four *α* levels. With the decrease of *α* (from 1.0 to 0), the uncertain band decreases too. That is, bigger uncertainties of inputs cause larger uncertainties of outputs. In fact, *α*=1.0 corresponds to the case that all parameters have crisp values. This means the plot of Fig. 9a is the same as the one given in Fig. 8. Besides, Fig. 10 gives a 3D plot of *hsf*_3__*hse* for the FCPN model, where we show the curve at each *α* level. This figure describes the effects of uncertainties. For comparison, we give another 3D plot of *hsf*_3_, illustrated in Fig. 11.

**Fig. 12:**
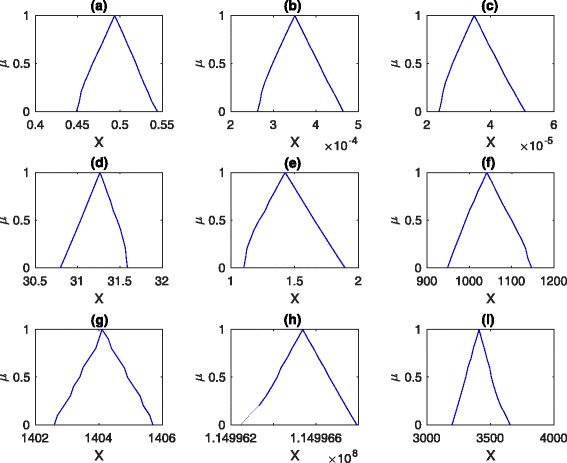
The membership functions of nine species at the simulation end time point. The same values of the kinetic parameters are used as those given in Fig. 9. **a***hsp*, **b***hsf2*, **c***hsf3*, **d***hse*, **e***hsf3_hse*, **f***hsp*, **g***hsp_hsf*, **h***mfp*, **i***prot*

Moreover, by composing the simulation results of all the *α*-cuts at the simulation end time point, we obtain the membership function of each output, which is illustrated in Fig. 12. From this figure, we can clearly observe the uncertain range of each output. For example, the uncertain range of *hsf*_3__*hse* is between 2.6×10^−4^ and 4.7×10^−4^. According to this, we can carefully check the effects of different uncertain parameters.

#### Simulative model checking.

Considering plenty of traces generated by the simulation of an FCPN model, we want to further check if these traces are similar or distinct in terms of their shape. By doing this, we may deduce those parameters that cause severe changes of the model. To address this issue, we apply PLTL model checking [16] to analyze traces from the FCPN model. From Fig. 8, we can see the trace has only one peak, so we want to check if all the traces have only one peak. Therefore, we can define the following two queries:

*P*_=?_[*F*((*d*[*Tr*]>0)&*F*((*d*[*Tr*]<0)))]

*P*_=?_[*F*((*d*[*Tr*]>0)&*F*((*d*[*Tr*]<0)&*F*((*d*[*Tr*]>0))))]

Here we use the function *d*(*species*) to get the derivative of the concentration of the species at each time point. The first query checks if there is a peak in a trace *Tr*, which is evaluated to true for all the traces of the FCPN model in Fig. 7. However, this query cannot answer the uniqueness of the peak. So we use the second query to check if there is a second peak, which is evaluated to false for all the traces. Of course, we can write more complicated queries to check more complex shapes of traces. For more details about simulative model checking, please refer to [16].

## Conclusions

In this paper, we present an FCPN approach for modeling and analyzing biological systems with uncertain kinetic parameters. An FCPN model is equivalent to a set of both FDEs and ODEs. Considering the fact that ODE/CPN modeling is widely used in the field of systems biology and the fact that uncertainties exist in many biological systems, we believe our approach could offer a good means to study uncertain biological systems.

In a next step, we will continue to explore the uncertain modeling issue in the field of systems biology, and then develop appropriate approaches for solving this issue. Specifically, we will concentrate on several typical biological systems and give some case studies with fuzzy features.
